# Upregulation of Wnt5a promotes epithelial-to-mesenchymal transition and metastasis of pancreatic cancer cells

**DOI:** 10.1186/1471-2407-13-496

**Published:** 2013-10-25

**Authors:** Haiji Bo, Shuhui Zhang, Li Gao, Ying Chen, Jing Zhang, Xuejiao Chang, Minghua Zhu

**Affiliations:** 1 Department of Pathology, Changhai Hospital, Second Military Medical University, Shanghai, China; 2 Department of Pathology, Yueyang Hospital, Shanghai University of Traditional Chinese Medicine, Shanghai, China

**Keywords:** Pancreatic cancer, Wnt5a, Epithelial-to-mesenchymal transition, Metastasis

## Abstract

**Background:**

Pancreatic cancer is one of the most lethal cancers worldwide. The aim of this study was to determine the expression pattern, clinical significance, and biological functions of Wnt5a in pancreatic cancer.

**Methods:**

Immunohistochemistry was performed to examine Wnt5a expression in 134 surgically resected pancreatic adenocarcinoma and adjacent normal pancreatic tissues. Associations of Wnt5a expression with clinicopathological factors and cancer-specific survival were analyzed. The effects of Wnt5a overexpression or silencing on the invasiveness and epithelial-to-mesenchymal transition (EMT) of pancreatic cancer cells were studied. Silencing of β-catenin by small interfering RNA was done to determine its role in the Wnt5a-mediated tumor phenotype.

**Results:**

The percentage of Wnt5a positive expression showed a bell-shaped pattern in pancreatic cancer tissues, peaking in well-differentiated carcinomas. The median cancer-specific survival was comparable between patients with positive versus negative expression of Wnt5a. Overexpression of Wnt5a promoted the migration and invasion of pancreatic cancer cells, whereas Wnt5a depletion had an inhibitory effect. In an orthotopic pancreatic cancer mouse model, Wnt5a overexpression resulted in increased invasiveness and metastasis, coupled with induction of EMT in tumor cells. Treatment with recombinant Wnt5a elevated the nuclear β-catenin level in pancreatic cancer cells, without altering the Ror2 expression. Targeted reduction of β-catenin antagonized exogenous Wnt5a-induced EMT and invasiveness in pancreatic cancer cells.

**Conclusion:**

Upregulation of Wnt5a promotes EMT and metastasis in pancreatic cancer models, which involves activation of β-catenin-dependent canonical Wnt signaling. These findings warrant further investigation of the clinical relevance of Wnt5 upregulation in pancreatic cancer.

## Background

Pancreatic cancer is a malignant tumor of the pancreas, with an estimated 277,000 new cases and 266,000 deaths annually worldwide [1]. Pancreatic cancer has an extremely poor prognosis. The 5-year survival rate of patients with pancreatic cancer is only about 4% [2]. Currently, surgical resection remains the only potentially curative treatment for localized tumors that are confined to the pancreas. Unfortunately, 80-85% of patients present with advanced unresectable disease at initial diagnosis [3]. Moreover, pancreatic cancer usually exhibits a poor response to most chemotherapeutic agents. Therefore, there is an urgent need to uncover the biological mechanisms contributing to development and progression of pancreatic cancer.

The lethal nature of pancreatic cancer is largely attributable to its propensity for early lymphatic invasion and distant metastasis. In general, metastasis involves a series of events, including loss of cell-cell adhesion, increased motility/migration, intravasation into blood and/or lymph vessels, disseminating through the circulation, extravasation, and colonization at distant sites [4]. Accumulating evidence suggests that epithelial-to-mesenchymal transition (EMT) plays an important role in tumor progression and metastasis in various solid tumors including pancreatic cancer [5,6]. During EMT epithelial cells undergo morphological changes and convert to a mesenchymal cell phenotype. Also, there are characteristic alterations in molecular markers, i.e., downregulation of epithelial adhesion molecule E-cadherin and upregulation of mesenchymal markers, such as vimentin and N-cadherin. Induction of EMT in pancreatic cancer cells is associated with increased migratory capacity and invasiveness [7,8].

Wnt proteins constitute a large family of secreted lipid-modified glycoproteins. The Wnt family is implicated in a variety of cellular processes, such as proliferation, apoptosis, differentiation, and migration [9]. Each family member exhibits unique expression patterns and distinct biological functions. Wnt signaling can be broadly divided into two categories: the canonical, β-catenin-dependent pathway and the non-canonical β-catenin-independent pathway [10]. Wnt5a has been identified as a non-canonical Wnt protein. Many studies have documented a crucial role for Wnt5a in cancer progression and metastasis, contributing to cancer cell migration and invasion [11,12]. Antibody-mediated suppression of Wnt5a activity results in the prevention of metastasis of gastric cancer cells [13]. However, conflicting results are obtained in different cellular contexts. Hansen et al. [14] reported that Wnt-5a can inhibit breast cancer cell migration in a CREB-dependent manner.

It has been documented that Wnt5a is upregulated during pancreatic carcinogenesis and mediates the migration and invasion of pancreatic cancer cells induced by the transcription factor CUTL1 [15]. The aim of this study was to determine the expression pattern and clinical significance of Wnt5a in pancreatic cancer and clarify how Wnt5a contributed to aggressive phenotypes of pancreatic cancer cells.

## Methods

### Tissue specimens

A total of 134 human pancreatic adenocarcinoma and adjacent normal pancreatic tissues were obtained from the Changhai Hospital Affiliated to Second Military Medical University (Shanghai, China), which were resected at this hospital between January 2002 and December 2004. Demographic and clinicopathological data of patients were collected from impatient medical records. Follow-up data were available in 45 patients of this cohort (34%). The median follow-up period was 15 months (range, 4–60 months). Tumor histological differentiation was defined by two pathologists, according to the World Health Organization classification [16]. In total, 30 cases were well differentiated, 83 moderately differentiated, and 21 poorly differentiated. Written informed consent was obtained from all patients and this study was approved by the Ethical Committee of Changhai Hospital of the Second Military Medical University.

### Cell culture and transfection

Human pancreatic cancer cell lines PANC-1 and BXPC-3 were obtained from Institute of Cellular Research, Chinese Academy of Science, Shanghai, China, and cultured in RPMI 1640 medium supplemented with 10% fetal bovine serum (FBS), 100 U/mL penicillin and 100 U/mL streptomycin (all from Invitrogen-Gibco, Carlsbad, CA, USA) in a 5% CO_2_ incubator at 37°C.

Wnt5a-expressing plasmid was constructed by subcloning the human Wnt5a cDNA into the pcDNA3.1 vector (Invitrogen, Carlsbad, CA, USA). Knockdown of endogenous Wnt5a expression in pancreatic cancer cells was achieved using small interfering RNA (siRNA) technology. The target sequence was 5'-GTTTTGGCCACTGACTGA-3'. The Wnt5a siRNA expression cassette was subcloned into the expression vector pcDAN6.2. Cell transfection was performed using the Lipofectamine 2000 Transfection Reagent, according to the manufacturer’s instruction (Invitrogen). To establish stable clones, transfected cells were selected with G418 (Invitrogen-Gibco) or blasticidin (Merck, Darmstadt, Germany). After selection for 2–3 weeks, single colonies were isolated and screened for Wnt5a expression by Western blot analysis.

β-Catenin and scrambled control siRNAs were purchased from GenePharma company (Shanghai, China). For assessment of the role of β-catenin in Wnt5a-mediated tumor phenotype, pancreatic cancer cells were transfected with β-catenin or control siRNA (20 nM) 24 h before the addition of recombinant human Wnt5a (200 ng; Abcam, Cambridge, MA, USA). After incubation for another 24 h, cells were tested for evidence of EMT and invasion ability.

### Western blot analysis

Cells were lysed with the lysis buffer containing a protease inhibitor mixture (Roche Applied Science, Mannheim, Germany) on ice for 30 min. Cytoplasmic and nuclear proteins were separately isolated using the Proteo JET Cytoplasmic and Nuclear Protein Extraction Kit, according to the manufacturer’s instructions (Fermentas, Burlington, ON, Canada). For dephosphorylation of Ror2 receptor, lysates were treated with 20 unit of calf intestinal alkaline phosphatase (CIAP; Promega, Madison, WI, USA) at 37°C for 1 h. Proteins were separated with 10% sodium dodecyl sulfate polyacrylamide gels and transferred onto polyvinylidene difluoride membranes (Bio-Rad, Hercules, CA, USA). The membranes were probed with specific antibodies and reactive proteins were detected using ECL chemiluminescence (Amersham Pharmacia Biotech, Piscataway, NJ, USA). Sources of antibodies and concentrations used were as follows: anti-Wnt5a (Abcam, Cambridge, MA, USA; 1:1000), anti-E-cadherin (Abcam; 1:1000), anti-vimentin (Abcam; 1:2000), anti-phosphorylated Ror2 (Abgent, San Diego, CA, USA; 1:500), anti-snail (Abcam; 1:100), anti-β-catenin (Abcam; 1:1000), anti-glyceraldehyde-3-phosphate dehydrogenase (GAPDH; Kangchen, Shanghai, China; 1:3000), and anti-lamin A/C (sc-20681, Santa Cruz Biotech, Santa Cruz, CA, USA; 1:1000). The intensity of each band was measured by densitometric analysis using the Quantity One software (Bio-Rad).

### Immunohistochemistry

Examination of the expression and distribution of Wnt5a in pancreatic cancer tissues was performed using the streptavidin-peroxidase-biotin immunohistochemical method. In brief, 4-μm paraffin-embedded sections were deparaffinized and rehydrated. Endogenous peroxidase activity was blocked by 3% hydrogen peroxide for 15 min. After antigen retrieval, sections were incubated with 5% serum to avoid the non-specific binding. The primary antibodies against Wnt5a, vimentin, and E-cadherin, each diluted in 1:50, were added onto the sections and incubated at 4°C overnight. The sections were then treated with biotinylated secondary antibodies, followed by incubation with streptavidin-horseradish peroxidase complex (Santa Cruz Biotech). Immunoreactivity was visualized with diaminobenzidine (Sigma-Aldrich, St. Louis, MO, USA). The sections were counterstained with hematoxylin. For blank controls, the primary antibody was omitted. For negative controls, the primary antibody was replaced by nonimmune serum.

The stained slides were scored independently by two pathologists blinded to clinical data. The proportion of positive tumor cells was scored as follows: 0 (≤10% positive tumor cells); 1 (11-24% positive tumor cells); 2 (25-50% positive tumor cells); 3 (51-75% positive tumor cells), and 4 (>75% positive tumor cells). Staining intensity was graded according to the following criteria: 1 (absent or weak staining); 2 (moderate staining) and 3 (strong staining). Staining index (SI) was calculated as the product of the proportion of positive tumor cells and staining intensity score. The cut-off value for distinguishing positive and negative Wnt5a expression was set as an SI of 3.

### Cell migration assay

Transfected PANC-1 and BXPC-3 cells were seeded onto fibronectin-coated 6-well plates in RPMI 1640 medium with 0.5% FBS. After 24 h, the cells were scratched using a sterile pipette tip, washed twice, and incubated in serum-free medium. The extent of scratch closure was quantified by measuring the area of the scratch before and 24 h after migration and results were expressed as percentage of wound closure.

### Transwell invasion assay

The Matrigel was diluted in 1:3 with ice-cold serum-free medium, added on the filters, and dried under a hood. RPMI 1640 medium with 10% FBS was applied to the lower chamber. Cells (2 × 10^5^) suspended in serum-free medium were seeded into the upper chamber and incubated at 37°C for 24 h. The cells on the upper surface of the filter were removed by wiping with a cotton swab. The filters were fixed and stained with crystal violet. The cells that penetrated to the lower surface of the filter were counted.

### Orthotopic pancreatic tumor model

Four-week-old nude mice (Crl:NU/NU-nuBR) weighing 14 to 16 g were obtained from Shanghai Laboratory Animal Center of Chinese Academy of Sciences (Shanghai, China). The animals were housed in a specific pathogen-free environment with a constant temperature of 25°C in 12-h light/12-h dark cycles and fed with a standard pellet diet and water ad libitium. All experiments involving animals in this study were conducted in accordance with the Guidelines for the Care and Use of Laboratory Animals of China. The protocol was approved by the Animal Care and Use Committee of Chinese Academy of Sciences (Permit number: No: Lacp0010).

Nude mice were anesthetized by peritoneal injection of chloral hydrate at 0.4 mg/g body weight. The abdomen of animals was opened via a 1-cm longitudinal incision in the left hypochondrium, and the pancreas was gently exteriorized. A total of 5 ×10^7^ cells per each cell line were injected into the pancreatic parenchyma nearby the hilum of the spleen. Afterwards the pancreas was relocated into the abdominal cavity. The abdomen was then closed with 6–0 absorbable vicryl sutures. Each group consisted of 3 animals. When the orthotopic tumors reached a size of 1 to 2 cm in the largest diameter, the mice were humanely killed by cervical dislocation. Organs with metastatic lesions were photographed. Xenograft tumors were resected, fixed in formalin, embedded in paraffin, and cut into sections. The sections were stained with hematoxylin and eosin (H&E) or immunostained with either anti-vimentin or anti-E-cadherin antibodies, as described above.

### Statistical analysis

The Chi-square test was performed to examine the associations of Wnt5a expression with clinicopathological factors. The end point of this study was cancer-specific death. Survival curves were generated using the Kaplan-Meier method and compared using the log-rank test. Differences in the means were determined using the Student *t*-test or one-way analysis of variance (ANOVA) followed by the Tukey test. A *P* value of <0.05 was considered statistically significant.

## Results

### Wnt5a expression in human pancreatic cancer tissues

**Figure 1:**
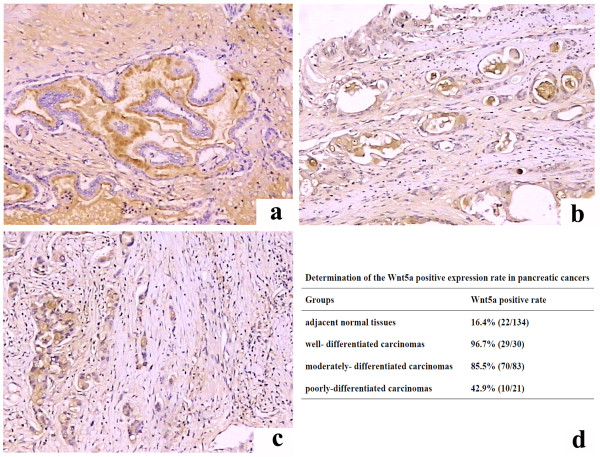
**Immunohistochemical analysis of Wnt5a expression in pancreatic cancer and adjacent normal tissues. (a-c)** Representative immunostaining of Wnt5a in well-differentiated **(a)**, moderately-differentiated **(b)**, and poorly-differentiated **(c)** pancreatic carcinomas. **(d)** Determination of the Wnt5a positive expression rate in adjacent normal pancreatic tissues and well-, moderately-, and poorly-differentiated pancreatic cancers.

Overall, 81.3% (109/134) of all pancreatic cancers showed positive expression of Wnt5a. The Wnt5a immunostaining intensity increased with the degree of tumoral differentiation (Figure 1a-c). In well-differentiated carcinomas, strong immunostaining for Wnt5a was detected at the glandular luminal border, whereas in poorly differentiated carcinomas the immunoreactivity was markedly reduced.

The positive expression rate of Wnt5a was 96.7% (29/30), 85.5% (70/83), and 42.9% (10/21) in well-, moderately-, and poorly-differentiated carcinomas, respectively (Figure 1d). In contrast, only 16.4% (22/134) of all adjacent normal tissues displayed positive staining for Wnt5a. Taken together, the Wnt5a protein expression exhibited a bell-shaped pattern in human pancreatic cancer tissues.

### Associations of Wnt5a expression with clinicopathological features and prognosis

The associations of Wnt5a expression with clinicopathological characteristics are summarized in Table 1. Wnt5a expression tended to be negatively associated with tumor histological grade (*P* < 0.001). However, no statistically significant relationships were found between Wnt5a expression and other clinicopathological factors, including gender, age, tumor location, tumor size, perineural invasion, pT classification, and lymph node metastasis.

**Table 1:** Associations of Wnt5a expression with clinicopathological characteristics in pancreatic cancer

**Variable**	**Category**	**n**	**Wnt5a negative**	**Wnt5a positive**	***P***
**Age(yr)**	≤60	70	16	54	0.192
	>60	64	9	55	
**Gender**	Male	85	18	67	0.324
	Female	49	7	42	
**Tumor location**^**b**^	Head	104	19	85	0.768
	Body/tail	29	6	23	
**Tumor size (cm)**^**c**^	≤2.0	25	3	22	0.559
	>2.0	93	18	75	
**Histological grade**	G1	30	1	29	<0.001^a^
	G2	83	13	70	
	G3	21	11	10	
**Perineural invasion**	Absent	25	7	18	0.252
	Present	109	18	91	
**pT**^**c**^	T1	25	3	22	0.319
	T2	31	7	24	
	T3	54	8	46	
	T4	8	3	5	
**Lymph node metastasis**^**d**^	Absent	24	7	17	0.147
	Present	83	13	70	

^a^*P* < 0.05 indicating significant differences.

^b^ Excluding 1 cases with unknown location of tumor.

^c^ Excluding 16 cases with unknown tumor size and pT classification.

^d^ Excluding 27 cases with unknown lymph node metastasis status.

**Figure 2:**
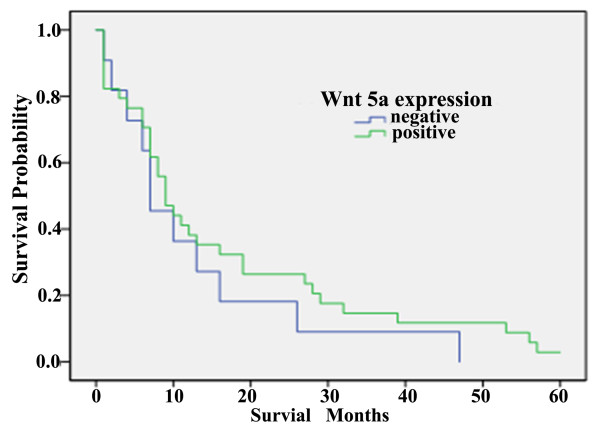
Kaplan–Meier survival curves in pancreatic cancer patients with and without Wnt5a expression.

The prognostic significance of Wnt5a expression in pancreatic cancer was evaluated using the Kaplan-Meier survival curve analysis (Figure 2). The patients with Wnt5a-positive tumors had a slightly higher median cancer-specific survival than those with negative Wnt5a expression (8.7 vs. 6.8 months); however, the difference was not statistically significant (*P* > 0.05).

### Wnt5a increases pancreatic cancer cells migration and invasiveness in vitro culture

**Figure 3:**
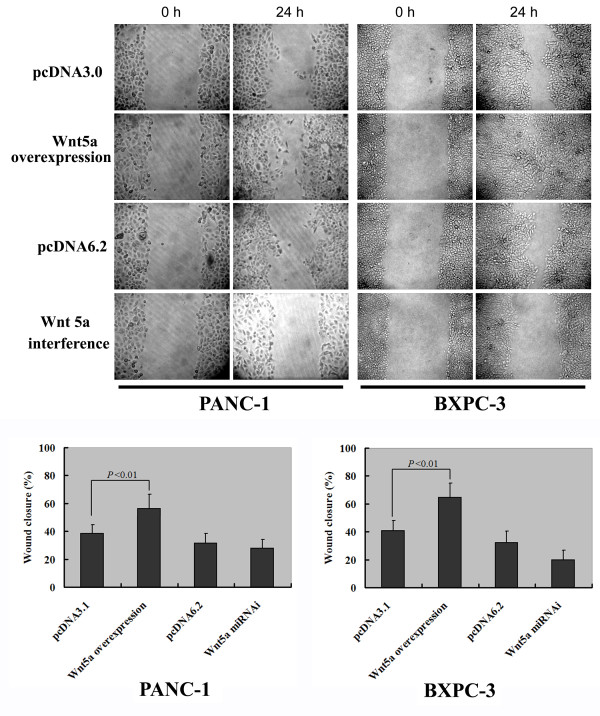
**Effect of Wnt5a on the migration of pancreatic cancer cells.** PANC-1 and BXPC-3 cells transfected with indicated plasmids were wounded and assessed for wound closure after 24 h. Representative images (top panels) of three independent experiments with similar results are shown. The bar graphs (bottom panels) depict the quantification of cell migration. Results are expressed as mean ± SD.

Next, we examined the biological functions of Wnt5a in pancreatic cancer. The scratch assay revealed that the percentage of wound closure at 24 h was significantly (P < 0.05) higher in Wnt5a-overexpressing pancreatic cancer cells than in empty vector-transfected cells (Figure 3). Moreover, siRNA-mediated silencing of Wnt5a profoundly reduced the migration of BXPC-3 cells, but did not affect the migration of PANC-1 cells (Figure 3).

**Figure 4:**
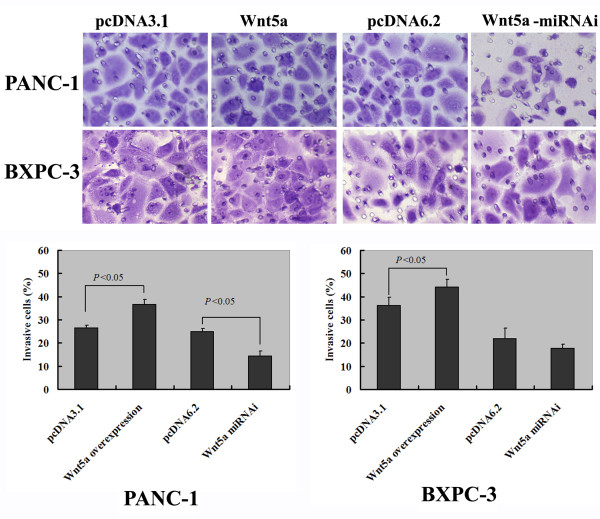
**Effect of Wnt5a on the invasiveness of PANC-1 and BXPC-3 cells transfected with indicated plasmids.** Transwell invasion assay was performed as described in *Methods*. Representative images of three independent experiments are shown in top panels. The bar graphs (bottom panels) show the average number of invaded cells per microscopic field (×200) for each condition.

Transwell invasion assay indicated that Wnt5a overexpression significantly (*P* < 0.05) promoted the invasiveness of PANC-1 and BXPC-3 cells by 40% and 28%, respectively (Figure 4). Wnt5a-depleted PANC-1 cells had a significantly (*P* < 0.05) lower invasive capacity than control siRNA-transfected counterparts (Figure 4). However, there was no significant difference in the invasion potential between Wnt5a-deficient and control BXPC-3 cells (*P* > 0.05).

### Wnt5a increases pancreatic cancer invasion and metastasis in vivo

**Figure 5:**
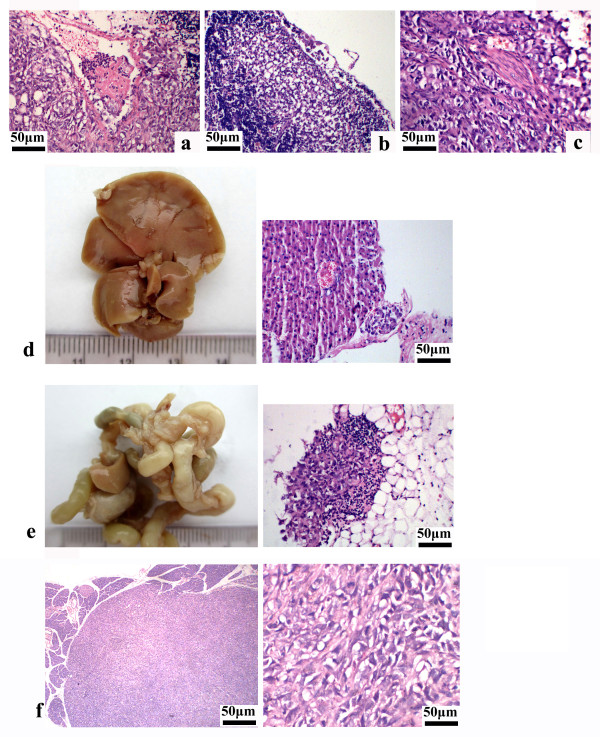
**Effects of Wnt5a on invasiveness and metastasis in an orthotopic pancreatic cancer mouse model.** Wnt5a-overexpressing or control pancreatic cancer cells were orthotopically injected into the pancreatic parenchyma of nude mice. Macroscopic and microscopic examination of metastases was done as described in *Methods*. Representative images of H&E staining showing vascular **(a)**, lymphatic **(b)**, and perineural **(c)** invasion of Wnt5a-overexpressing PANC-1 cells. Scale bar = 50 μm. Macroscopic appearance of the liver **(d)** and mesentery **(e)** showing prominent metastasis in the mice injected with Wnt5a-overexpressing PANC-1 cells. **(f)** Representative micrograph showing H&E-stained sections of control tumors from empty vector-transfected PANC-1 cells. Scale bar = 50 μm.

Using an orthotopic mouse model of pancreatic cancer, we further assessed the effect of Wnt5a on pancreatic cancer invasion and metastasis in vivo. Wnt5a-overexpressing PANC-1 and BXPC-3 cells orthotopically injected into nude mice showed vascular, lymphatic, and perineural invasion, as determined by pathological examination (Figure 5a-c). Moreover, such cells formed metastatic tumors in multiple sites in all recipient mice, including the liver (Figure 5d) and mesentery (Figure 5e). Regarding the empty vector-transfected cells, no metastasis was found when they were inoculated into nude mice (Figure 5f).

### Wnt5a induces EMT in pancreatic cancer cells

**Figure 6:**
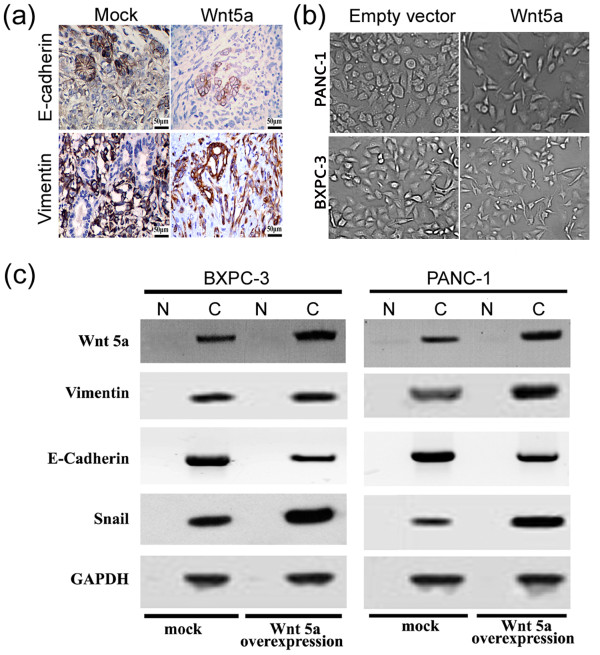
**Induction of EMT in Wnt5a-overexpressing pancreatic cancer cells. (a)** Immunohistochemical analysis of vimentin and E-cadherin, two EMT markers, in orthotopic pancreatic tumors. Representative immunostaining images showing increased expression of vimentin and decreased expression of E-cadherin in Wnt5a-overexpressing PANC-1 tumors, compared to control tumors derived from empty vector-transfected PANC-1 cells. Scale bar = 50 μm. **(b)** Cell morphology of PANC-1 cells transfected with Wnt5a-overexpressing plasmid or empty vector under a phase-contrast microscope. Magnification, ×200. **(c)** Western blot analysis of the expression of indicated EMT markers in mock- and Wnt5a-transfected PANC-1 and BXPC-3 cells. Representative blots of three independent experiments with similar results are shown. GAPDH was used as an internal control. N: nuclear fraction; C: cytoplasmic fraction.

We next checked the effect of Wnt5a on the EMT of pancreatic cancer cells. Immunohistological analysis demonstrated that Wnt5a-overexpressing tumors exhibited increased expression of vimentin and decreased expression of E-cadherin, indicative of the occurrence of EMT, compared to control tumors derived from empty vector-transfected pancreatic cancer cells (Figure 6a). The induction of EMT was further confirmed in pancreatic cancer cells transfected with Wnt5a-expressing plasmids. As shown in Figure 6b, overexpression of Wnt5a induced a mesenchymal spindle-like morphology in PANC-1 cells under a phase-contrast microscope. Western blot analysis further confirmed that Wnt5a-overexpressing pancreatic cancer cells undergo an EMT, as evidenced by an increase in the expression of vimentin and snail and a concomitant reduction in the E-cadherin expression (Figure 6c).

### Wnt5a activates β-catenin signaling in pancreatic cancer cells

**Figure 7:**
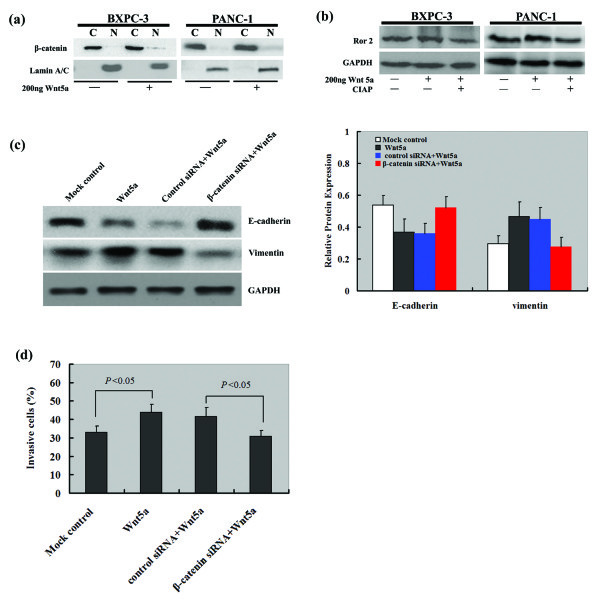
**Western blot analysis of the expression of β-catenin and Ror2 in PANC-1 and BXPC-3 cells with indicated treatments. (a)** Treatment of pancreatic cancer cells with recombinant Wnt5a (200 ng) resulted in an increase in the nuclear β-catenin level. Lamin A/C was used as a nuclear internal control. N: nuclear fraction; C: cytoplasmic fraction. **(b)** The Ror2 protein level of pancreatic cancer cells remained unchanged in the presence of recombinant Wnt5a with or without CIAP. GAPDH was used as an internal control. CIAP, calf intestinal alkaline phosphatase. **(c)** PANC-1 cells were transfected with β-catenin or control siRNA 24 h before exposure to recombinant Wnt5a. After incubation for another 24 h, Western blot analysis was performed to examine the protein expression of indicated EMT markers. The bar graph shows the results of densitometric analysis. **(d)** Transwell invasion assay was performed to test the invasiveness of PANC-1 cells transfected with β-catenin or control siRNA 24 h before treatment with recombinant Wnt5a. The bar graph represents the data from three independent experiments.

Western blot analysis revealed that exposure to recombinant Wnt5a raised the nuclear level of β-catenin without altering the total level of the protein, in both PANC-1 and BXPC-3 cells (Figure 7a), indicating a translocation of β-catenin from the cytoplasm to the nucleus. In contrast, Wnt5a treatment had no influence on the expression and phosphorylation of Ror2 (Figure 7b). Notably, siRNA-mediated silencing of β-catenin blocked recombinant Wnt5a-induced acquisition of the mesenchymal phenotype in pancreatic cancer cells (Figure 7c). Moreover, depletion of β-catenin reversed the promotion of cell invasion by exogenous Wnt5a, resulting in about 74% reduction in the invasiveness of tumor cells relative to control siRNA-transfected cells (Figure 7d).

## Discussion

Wnt5a has been found to be upregulated in several solid tumors, such as gastric cancer [17] and skin cancer [18]. Our data confirm the previous finding that there is an elevation in Wnt5a expression in pancreatic cancer tissues compared to normal pancreatic tissues [15]. Moreover, we found that Wnt5a expression intensity increased with the differentiation degree of tumors. Regarding the expression extensity in pancreatic cancer tissues, Wnt5a showed a bell-shaped expression pattern with a significant peak in well-differentiated carcinomas. Such expression pattern has also described in other human cancers. Kremenevskaja et al. [19] reported that Wnt5a shows a low to absent staining in normal thyroid tissues, strong positive staining in differentiated thyroid carcinomas, but a complete loss in all anaplastic tumors. Similarly, low to undetectable levels of Wnt5a are present in hepatocellular carcinoma (HCC) and normal liver tissues, whereas strong immunostaining was seen in chronic hepatitis, cirrhosis, and dysplastic liver cells [20]. The bell-shaped expression pattern suggests that Wnt5a may play a distinct role in different cellular contexts. Indeed, Wnt5a acts as a stimulator of metastasis in gastric cancer cells [13], but as a suppressor in breast cancer cells [14].

Metastasis remains a major cause of morbidity and mortality in cancer patients. Wnt5a is capable of regulating numerous biological events associated with metastasis. Cai et al. [21] reported that siRNA-mediated silencing of Wnt5a in breast cancer cells results in increased invasiveness, whereas overexpression of this gene suppresses breast cancer cell invasion. Wnt5a contributes to gastric cancer cell dissemination to the liver through upregulation of laminin gamma 2 [22]. In agreement with the report by Ripka et al. [15], our in vitro evidence indicates that Wnt5a acts as a potent activator of tumor cell migration and invasion in pancreatic cancer. However, such stimulating effects were not consistently observed in both PANC-1 and BXPC-3 cells. The varying consequences of manipulating Wnt5a further reflect the context dependence of Wnt5a function. Using an orthotopic pancreatic cancer model, we demonstrated that Wnt5a-overexpressing cancer cells formed metastatic tumors at multiple sites, whereas control cells failed to metastasize. These results underscore an important role for Wnt5a in pancreatic cancer invasion and metastasis.

Induction of EMT has been associated with increased cancer metastasis and aggressive clinical behaviors [23]. We found that compared to adjacent normal tissues, Wnt5a-overexpressing tumor tissues had elevated expression of vimentin and reduced expression of E-cadherin, indicating the presence of EMT. In vitro studies further confirmed the induction of EMT in pancreatic cancer cells by Wnt5a, as evidenced by increased expression of vimentin and reduced expression of E-cadherin. The transcription factor snail has been identified as a critical stimulator of EMT. Many studies suggest that snail represents a converging point of several signaling pathways including Wnt signaling, all eventually leading to EMT [24,25]. Yook et al. [25] reported that snail is implicated in Wnt-1-induced EMT in MCF-7 cells. Our data demonstrated that enforced expression of Wnt5a resulted in increased snail protein expression in pancreatic cancer cells, suggesting an involvement of snail in Wnt5a-induced EMT of pancreatic cancer cells.

The interactions of Wnt ligands with transmembrane receptors known as Frizzled (Fzd) and co-receptors initiate the activation of intracellular signaling pathways. Wnt5a can exert its biological effects through the canonical or non-canonical Wnt signaling pathway, largely depending on the availability of specific receptors [26]. It has been reported that addition of recombinant Wnt5 led to increased nuclear β-catenin in PANC-1 cells as well as a significant increase in TCF/LEF-dependent reporter activity [15], indicative of an activation of the canonical signaling pathway. Consistent with this study, our data showed a promotion of β-catenin nuclear translocation in both PANC-1 and BXPC-3 cells by exposure to recombinant Wnt5a. Moreover, we showed that the protein level Ror2 receptor remained unchanged after the treatment with recombinant Wnt5a, suggesting no activation of the non-canonical Wnt signaling pathway. Mounting evidence indicates a critical role for the β-catenin signaling in the pathogenesis of pancreatic cancer [27,28]. Wang et al. [27] documented that expression of the ataxia-telangiectasia group D complementing (ATDC) gene increases β-catenin levels in pancreatic cancer, consequently facilitating tumor growth and metastasis. Kobayashi et al. [28] reported that degradation of β-catenin mediates galectin-3 silencing-induced suppression of pancreatic cancer cell migration and invasion. Activation of the β-catenin signaling is linked to initiation of EMT during pancreatic cancer [29]. In agreement with these findings, our in vitro data demonstrated that targeting β-catenin antagonized Wnt5a-induced EMT and invasiveness, indicating an essential role for the β-catenin signaling in Wnt5a-mediated tumor aggressiveness.

Although the in vitro evidence indicated a master role of Wnt5a in inducing aggressive tumor phenotypes, the prognostic impact of Wnt5a expression in pancreatic cancer appears not to be of significance. We found that there was no significant difference in the cancer-specific survival of patients with Wnt5a positive vs. negative pancreatic tumors. Such inconsistency between the biological and clinical findings remains to be further clarified. The possibility can not be excluded that tumoral Wnt5 expression may have significant prognostic implications in a subgroup of pancreatic cancer patients.

## Conclusion

In conclusion, our data confirm an upregulation of Wnt5a in pancreatic cancer, especially well-differentiated carcinomas. This Wnt protein plays an important role in regulating EMT and metastasis in pancreatic cancer models. Such tumor aggressiveness conferred by Wnt5a is mediated through activation of β-catenin-dependent canonical Wnt signaling. However, further studies are certainly needed to clarify the real clinical value of Wnt5a.

## Competing interests

The authors declare that they have no competing interests.

## Authors’ contributions

MZ designed research; HB, SZ, LG, YC, and JZ performed research; HB and XC analyzed data, HB, SZ and MZ wrote the paper. All authors read and approved the final manuscript.

## Pre-publication history

The pre-publication history for this paper can be accessed here:

http://www.biomedcentral.com/1471-2407/13/496/prepub
